# Antisocial Personality Problems in Emerging Adulthood: The Role of Family Functioning, Impulsivity, and Empathy

**DOI:** 10.3390/brainsci11060687

**Published:** 2021-05-23

**Authors:** Eleonora Marzilli, Luca Cerniglia, Silvia Cimino

**Affiliations:** 1 Department of Dynamic and Clinical Psychology, Sapienza, University of Rome, Via degli Apuli, 1, 00185 Rome, Italy; eleonora.marzilli@uniroma1.it; 2 Faculty of Psychology, International Telematic University Uninettuno, 00186 Rome, Italy; l.cerniglia@uninettunouniversity.net

**Keywords:** antisocial personality problems, emerging adulthood, family functioning, impulsivity, empathy

## Abstract

International research has evidenced the key role played by adults’ and adolescents’ family functioning, impulsivity, and empathy in antisocial personality problems. To date, no study has assessed the complex interaction between these variables during emerging adulthood. This study aimed to explore the possible interplay between antisocial personality problems, the quality of family functioning, impulsivity, and empathetic problems in a community sample of 350 emerging adults. Descriptive, correlational, hierarchical regression, and mediation analyses were performed, controlling relevant socio-demographic variables. Results showed a predictive effect of parental behavioral control, motor impulsivity, and empathetic concern in antisocial personality problems. Moreover, motor impulsivity and empathetic concern partially mediated the relationship between parental behavioral control and emerging adults’ antisocial personality problems. This study supports the recent evidence on the complex relationship between individual and relational protective and risk factors involved in antisocial personality problems during emerging adulthood, with important implications for their intervention treatments.

## 1. Introduction

Antisocial personality disorder (ASPD) is a severe personality disorder characterized by a pervasive pattern of disregarding or violating others’ rights, often without showing interest in the feelings of others [1]. Specifically, according to the Diagnostic and Statistical Manual (DSM-5) [2], diagnostic criteria for ASPD include a set of seven criteria referring to both personality-oriented and behavior-focused indicators, including failure to conform to social norms; impulsivity; consistent irresponsibility; irritability and aggressiveness; and lack of remorse for having hurt, mistreated, or stolen from another. Moreover, to be diagnosed with ASPD, the subject must be at least 18 years old and must have had conduct disorder since at least the age of 15 [2]. However, personality and behavioral symptoms of ASPD are not uncommon in the general population [3], especially during adolescence and young adulthood, occurring in subclinical forms that do not satisfy the criteria to pose a diagnosis of ASPD [4]. This phenomenon represents a major public policy and health concern [5], due to its high rates of prevalence (more than 10% among youths) [6,7,8], and the recent increase in its global incidence [9]. In this field, although there is a wide range of studies that have examined antisocial personality problems among the adolescent population [10,11,12,13], far fewer studies have focused on this topic in the transition from adolescence to adulthood [14,15,16,17].

The developmental period of “emerging adulthood” (between the ages of 18 and 25) [18] is a developmental stage characterized by multiple challenges related to identity explorations and transitions in social roles; behavioral styles and personality traits emerge in this stage, with stability over time [19]. Thus, this critical window of maturation represents a key stage for studying personality difficulties involving antisocial personality problems. Indeed, although it has been underlined that antisocial behaviors generally have a peak of increase during late adolescence, with a decline in the course of emerging adulthood [20], there are individual differences in trajectories of antisocial personality problems [21,22,23]. Some youths, defined as “adolescence-limited” antisocials, show antisocial behaviors and callous–unemotional traits in late adolescence but tend to desist during emerging adulthood. In other cases, individuals may show persistent antisocial problems from adolescence to adulthood, a subtype of antisocials named “life-course persistent”. Finally, the presence of “late-onset” antisocials has also been evidenced, which manifest antisocial tendencies from emerging adulthood [17,21,22,23]. Consequently, it is important to identify risk and protective factors associated with antisocial personality problems in emerging adulthood, to implement the knowledge of underpinning mechanisms related to their persistent and/or late onset and to guide preventive programs and post-onset interventions [24]. The Developmental Psychopathology theoretical framework suggests considering the role played by multiple levels of analyses [25], from personality traits of vulnerability to environmental factors which may promote or mitigate antisocial personality problems in this developmental stage [26].

In this field, some studies have shown that social role changes occurring during emerging adulthood, including the presence of romantic relationships and employment, may exert a protective role on desistance from problem behaviors [27,28], with a significant increase in prosocial activities and a decrease in antisocial attitudes [29]. Despite these potential benefits, in the last decade, social and economic changes have resulted in a delay in the acquisition of these traditional adult social roles to which emerging adults increasingly tend to have access around age 30 [30], with a consequent increase in staying in the family home [31,32,33]. Moreover, some authors have suggested that previous parental influence could be internalized and continue to affect emerging adults’ psychological well-being even if the youth lives outside the family home [15,34]. Consequently, parental support and the quality of family functioning continue to assume a central source of reference and influence for early adults [30] and may represent a key protective and/or risk factor for the involvement in antisocial behaviors and personality problems [14,16]. In particular, international research has shown that the lack of parental warmth, emotional support, and behavioral control are associated with a wide range of youth’s externalizing problems, including aggression [35,36], addictive behaviors [37,38,39], and risk-taking [40,41]. Recently, the same associations have been reported also with antisocial traits and delinquency [42,43,44,45], but focusing almost exclusively on adolescent samples. Only a few studies have explored the association between family functioning and ASPD symptoms among the emerging adulthood population [14,15,16,17,46], although the important role played by emerging adults’ family functioning for their psychological well-being has been widely shown [33,47]. Regarding individual vulnerability factors, the presence of specific personality traits can further promote the onset or maintenance of youths’ antisocial personality problems [48,49]. Impulsivity and low empathy are two of the main personality dimensions most frequently associated with ASPD symptoms among emerging adults, both in clinical samples and in the general population [48,49,50,51,52,53,54,55,56]. Impulsivity traits may be defined as a predisposition to rapid, unplanned reactions to internal or external stimuli without considering the negative consequences of these reactions for the individual or others [57]. On the other hand, an emerging adult who manifests low empathy may have difficulties in understanding other’s feelings and disapproval, as well as the impact of their negative behaviors on others [58]. Although these personality characteristics are part of the DSM-5 diagnostic criteria for ASPD [2], a key role of impulsivity and low empathy in predicting personality disorder symptoms [48,49,54,59], including ASPD symptoms [48,49,54,55,56], has been shown. However, impulsivity and empathy are multidimensional constructs, but only a few studies have explored which specific dimension of these personality traits may be more predictive of antisocial personality problems during emerging adulthood [60,61,62,63]. 

Overall, a growing body of research has evidenced that young adults’ perception of a poor family functioning, low levels of empathy, and high impulsivity traits may exert a significant contribution to antisocial personality problems [14,15,16,17,46,48,54,55,56]. Interestingly, on the relationship between these risk factors, a predictive role of worse family functioning on both impulsivity problems [64,65,66] and low empathy [42,67] has been evidenced. Moreover, recent findings have shown that these personality traits mediated the relationship between a poor quality of family functioning and both psychological difficulties [39,68] and personality disorder symptoms [66]. In the field of antisocial personality problems, a recent study by Álvarez-García et al. [44] has found that impulsivity and empathy mediated the relationship between family functioning and antisocial behaviors among the adolescent population. However, despite this emerging evidence, to our best knowledge, no study has yet explored the possible mediation role played by empathy and impulsivity problems on the relationship between emerging adults’ perception of their family functioning and antisocial personality problems. 

**Figure 1:**
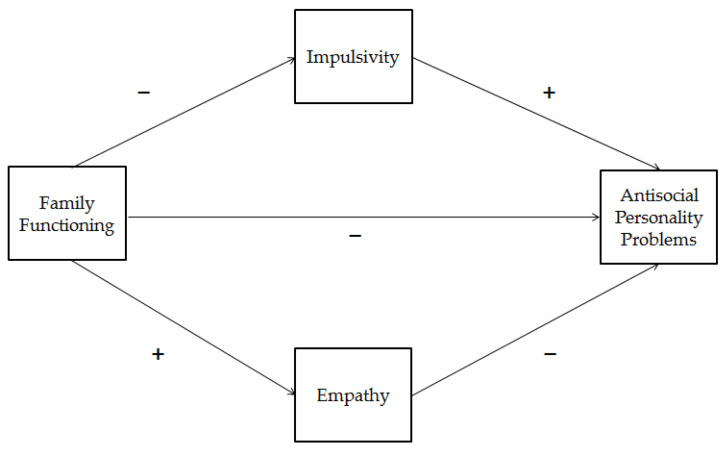
Conceptual model. + indicates positive statistical relationships; − indicates negative statistical relationships.

Based on these premises and literature gaps, the present study aimed to explore, in a community sample of male and female emerging adults, the complex relationship between antisocial personality problems and family functioning, impulsivity, and empathy. Specifically, we hypothesized that good quality of emerging adult’s family functioning and an adequate level of empathy had a protective effect on antisocial personality problems, whereas impulsivity problems had a risk effect. Moreover, we hypothesized that the relationship between family functioning and antisocial personality problems could be mediated by impulsivity and empathy. Figure 1 shows the conceptual model.

## 2. Materials and Methods

### 2.1. Sample Recruitment and Procedure

Over a period of 1 year, 443 emerging adults ranging in age from 18 to 25 years were recruited via notices posted on online psychology research websites and on social media. Prior to taking part in the study, all youths completed the written informed consent and gave their agreement to participate. The study was approved by the Ethical Committee of the Department of Dynamic and Clinical Psychology at Sapienza University of Rome (protocol N. 142/2019), in accordance with the Declaration of Helsinki. All participants who accepted to take part in the study filled out an anonymous self-completed online survey. First, participants completed an ad hoc questionnaire regarding sociodemographic data (i.e., age, gender, relationship status, living setup, educational level, and employment status). Then, self-report questionnaires for the assessment of antisocial personality traits, family functioning, impulsivity, and empathy (described below) were administered. 

### 2.2. Measures

#### 2.2.1. Assessment of Antisocial Personality Problems of Emerging Adults 

The Adult Self-Report (ASR) [69] is a 126-item self-report questionnaire assessing the psychological functioning of adults (ages 18–59). Items are rated on a 3-point Likert scale (from 0 = “not true” to 2 = “very true”). The ASR provides scores for eight syndrome scales (anxious/depressed, withdrawn, somatic complaints, thought problems, attention problems, aggressive behavior, rule-breaking behavior, and intrusive behavior) and six DSM-oriented scales (depressive problems, anxiety problems, somatic problems, avoidant personality problems, attention-deficit/hyperactivity problems, and antisocial personality problems). For the aim of this study, we used the scores of antisocial personality problems of the DSM-oriented scales. Specifically, the 20 items of the Antisocial Personality Problems scale refer to the symptoms of ASPD described in the DSM-5 [2]. Coherently, some items evaluate personality features of ASPD (e.g., “I blame others for my problems “, “I don’t feel guilty after doing something I shouldn’t have done”, “I have a hot temper”), while others explore behavioral aspects of the disorder (e.g., “I damage or destroy things of others”, “I lie or cheat”, “I physically attack people”). The scores of the 20 items were summed to compute the ASR Antisocial Personality Behavior. Achenbach and Rescorla [69] recommended using raw scores for analyses to avoid problems associated with censored measures. Consequently, as also suggested by other studies in the field [70], raw scores were used in the statistical analyses. The ASR showed good reliability and validity [69]. In the current sample, the ASR showed good internal coherence (Cronbach alpha = 0.82). 

#### 2.2.2. Assessment of Family Functioning of Emerging Adults 

The Family Assessment Device (FAD) [71,72] is a 60-item self-report questionnaire that was developed to measure various aspects of family functioning. Items are evaluated on a 4-point scale (from 1 = “strongly agree” to 4 = “strongly disagree”) and allow measuring six dimensions of the McMaster Model of Family Functioning: (1) Problem Solving (PS), which addresses the family’s ability to solve problems (e.g., “We resolve most emotional upsets that come up”); (2) Communication (COM), which evaluates whether communication between the family members is clear and direct or vague and indirect (e.g., “We are frank with each other”); (3) Roles, which addresses the issue of how roles and responsibilities are allocated among the family members (e.g., “We discuss who is to do household jobs”); (4) Affective Responsiveness (AR), which addresses ability of the family members to respond to a range of situations with appropriate quality and amount of emotion (e.g., “We do not show our love for each other”); (5) Affective Involvement (AI), which assesses how family members experience interest and involvement with each other (e.g., “If someone is in trouble, the others become involved too”); and (6) Behavioral Control (BC), which evaluates whether the family has norms or standards governing individual behavior and responses to emergency situations (e.g., “We have rules about hitting people”). Psychometric properties showed a good validity, reliability, and internal consistency [71,72]. In this study, the internal consistency of the six subscales was also adequate (Cronbach α = 0.78–0.92).

#### 2.2.3. Assessment of Impulsivity of Emerging Adults 

The Barratt Impulsiveness Scale 11 (BIS-11) [73], is a self-report questionnaire for the assessment of impulsive behaviors. It is composed of 30 items that are assessed on a 4-point scale (from 1 = ‘‘never–rarely’’ to 4 = ‘‘almost always–always’’). The BIS-11 allows obtaining a score on three main scales: (a) Attentional Impulsivity (inability to focus attention or concentrate, e.g., “Am a steady thinker”); (b) Motor Impulsivity (acting without thinking, e.g., “spend more than earn”); (c) Non-planning Impulsivity (lack of future orientation or forethought, e.g., “plan for job security”). Higher scores (i.e., >75) indicate an impulse-control disorder, while scores between 70 and 75 points indicate pathological impulsivity. Psychometric proprieties of the Italian version [74] showed good qualities (Cronbach’s alpha = 0.79 and test–retest reliability r = 0.88). Reliability values in the current sample were as follows: Attentional Impulsivity = 0.79, Motor Impulsivity = 0.88, and Non-planning Impulsivity = 0.89.

#### 2.2.4. Assessment of Empathy of Emerging Adults 

The Interpersonal Reactivity Index (IRI) [75,76,77] is a 28-item self-report measuring separate but intercorrelated components of empathy. Items are rated on a 5-point scale (from 1 = “Does not describe me well” to 5 = “Describes me very well”) which allows obtaining a score of four dimensions of empathy: (1) Perspective Taking (PT), which refers to the reported tendency to spontaneously adopt the psychological point of view of others in everyday life (e.g., “I try to look at everybody’s side of a disagreement before I make a decision”); (2) Empathic Concern (EC), which refers the tendency to experience feelings of sympathy and compassion for unfortunate others (e.g., “I often have tender, concerned feelings for people less fortunate than me”); (3) Personal Distress (PD), which assesses the tendency to experience severe discomfort in response to extreme distress in others during a tense emotional situation (e.g., “In emergency situations, I feel apprehensive and ill-at-ease”); (4) Fantasy (FS), which measures the tendency to imaginatively transpose oneself into fictional situations (e.g., “I daydream and fantasize, with some regularity, about things that might happen to me”). The IRI showed good psychometric properties [78], as did the Italian version [77]. In the present study, the internal consistency of the four dimensions was also adequate (Cronbach α = 0.81–0.89).

### 2.3. Statistical Analyses

Preliminary statistical analyses were conducted using descriptive statistics (reliability of the measures, frequencies, and mean scores). Then, Pearson’s correlation analyses were carried out to determine initial correlations between study variables. Based on significant correlations, we conducted hierarchical multiple regression analyses to identify the main effects of family functioning, impulsivity, and empathy on emerging adults’ antisocial problems, controlling for relevant demographic factors (i.e., age, gender, romantic relationship status, living status, level of education, and employment status). Finally, parallel mediation analyses were conducted to test whether impulsivity and empathy mediated the effect of family functioning on emerging adults’ antisocial personality problems. The covariates that showed a significant effect in regression analyses were also inserted as covariates in the mediation analyses. To this end, we used Hayes’s PROCESS macro [79] (Model 4), which provides coefficient estimates for total, direct, and indirect effects of variables using ordinary least squares regression. Indirect (i.e., mediating) effects were evaluated with 95% bias-corrected confidence intervals (CIs) based on 10.000 bootstrap samples. When a CI does not include zero, it indicates that the effect is significant at α = 0.05. All analyses were performed using IBM SPSS software 25.0.

## 3. Results

### 3.1. Sample Characteristics 

From the total sample, emerging adults that did not complete the assessment procedure (N = 46), reported a psychopathological diagnosis and/or physical disabilities (N = 24), or were following psychological and/or psychiatric treatment (N = 23) were excluded. The final sample consisted of N = 350 emerging adults (52.6% females) with average age of 22.16 years (DS = 2.18). The minority (38%) was single and 60% lived within the family. Participants most often reported their highest level of education being high school (44.3%) or more than high school (47.1%), and the vast majority were students without a job (30.6%) or part-time employed (24.9%). Table 1 shows the complete description of the sample demographic characteristics.

### 3.2. Bivariate Associations between Study Variables

Results of Pearson’s correlation analyses showed that the score of antisocial personality problems was significantly associated with all dimensions of FAD, except for problem solving and communication; with all dimensions of IRI, except for PD; and with all dimensions of BIS-11. Moreover, impulsivity and empathy were related to many of the FAD dimensions, allowing for the possibility that the relationship between family functioning and antisocial personality problems may be mediated by impulsivity and empathy (Table 2). 

### 3.3. Main Effects of Family Functioning, Impulsivity, and Empathy on Antisocial Personality Problems

Based on significant correlations, hierarchical multiple regression analyses were conducted to investigate whether family functioning, impulsivity, and empathy were predictive of levels of antisocial personality problems. After controlling for covariates, a significant negative effect of the dimension of behavioral control of FAD was found for early adults’ antisocial personality problems. Moreover, empathic concern was significantly negatively associated with antisocial personality problems, and a significant positive effect of motor impulsivity was also found. This model explained 64% of the variance (Table 3). 

### 3.4. Emerging Adults’ Impulsivity and Empathy as Mediators of the Relationship between Family Functioning and Antisocial Personality Problems

**Figure 2:**
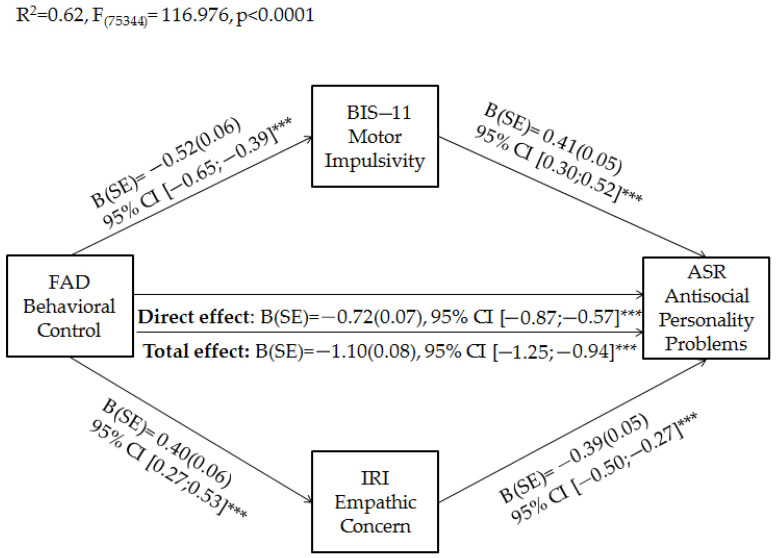
Parallel mediation of motor impulsivity and empathic concern on the relationship between parental behavioral control and emerging adults’ antisocial personality problems. *** *p* < 0.001.

Finally, based on significant predictive relationships that emerged in regression analyses, parallel mediation analyses were conducted to verify whether the relationship between behavioral control perceived by early adults in their family functioning and their own levels of antisocial personality problems was mediated by their motor impulsivity and empathic concern problems. Mediation analyses were adjusted for covariates that we found significantly related to antisocial personality problems in previous analyses (i.e., relationship status and occupation). Results of parallel mediation analyses showed that the total effect of behavioral control on emerging adults’ antisocial personality problems was significant. Moreover, behavioral control was negatively associated with motor impulsivity, which in turn was positively related to antisocial personality problems. On the other hand, behavioral control was positively related to empathic concern, which in turn was negatively associated with antisocial personality problems. When considering the effects of mediators, the direct effect of behavioral control on antisocial personality problems remained significant. Overall, the model explained 65% of the variance in emerging adults’ antisocial personality problems (Figure 2).

Regarding indirect effects, as possible to see in Table 4, the indirect paths via empathetic concern and motor impulsivity were significant. However, the coefficient of direct effect was larger than indirect effects, indicating a partial mediation.

## 4. Discussion

This study aimed to further increase the knowledge on protective and risk factors involved in antisocial personality problems in emerging adulthood. To this end, we have chosen to focus on relational and individual variables (i.e., family functioning, impulsivity, and empathy) that previous literature on adolescent and adult populations has indicated as significant predictors of both personality [48,49,54,55,56] and behavior features [44,45,52,80,81] of antisociality. In addition, we tested a conceptual framework that took into consideration the complex relationships between these variables, considering both the direct effect of family functioning and indirect effects via emerging adults’ impulsivity and empathy. Overall, our results are in accordance with our hypothesized model (Figure 1). 

In particular, hierarchical regression analyses confirmed a significant role of some specific dimensions of the emerging adult’s family functioning, impulsivity, and empathy on his/her antisocial personality problems. Specifically, after controlling relevant socio-demographic factors, the results showed a predictive effect of parental behavioral control, motor impulsivity, and empathetic concern, accounting for 64% of the variance in antisocial personality problems. High levels of parental behavioral control and empathetic concern were associated with low levels of antisocial problems, whereas high motor impulsivity problems had a positive effect. These results are in line with previous studies on individual personality traits, which have shown the central role played by impulsivity and empathy on the development of ASPD symptoms [48,54,55,56]. The association between impulsivity and antisocial personality problems is not surprising given that the Diagnostic and Statistical Manual of Mental Disorders (DSM-5) [2] also indicates the presence of impulsivity as one of the main criteria of ASPD. However, recent evidence has shown the importance of investigating which specific dimension of impulsivity can place the individual at a greater risk of antisocial personality problems [60,61,62,63]. Our results showed that only the motor dimension of impulsivity (defined as acting on the spur of the moment) was positively associated with antisocial personality problems. These findings are in line with the studies by Umut et al. [61] and Urben et al. [62] that found higher levels of motor impulsivity among individuals at risk and/or with a diagnosis of ASPD than in those without it. Interestingly, it has been recently evidenced that motor impulsivity may be implicated in the recidivism of antisocial behaviors [63]. In accordance with previous studies, our findings suggested that the behavioral aspect of impulsivity may be more predictive of antisocial personality problems in emerging adulthood. 

Moreover, our results showed that emerging adults’ antisocial personality problems were negatively predicted by high levels of empathetic concern, an affective dimension of empathy which refers to the ability to experience another’s feelings. These findings are in line with previous studies that have evidenced a close relationship between empathy and prosocial behaviors [82,83], suggesting a protective effect on behavioral aspects of antisocial personality problems. Conversely, empathy impairment, especially of the affective component, was be found in association with a higher risk of ASDP symptoms [84,85,86]. At the same time, experimental studies have revealed lower levels of physiological reactivity responses to social distress cues [87], as well as lower levels of neural response to viewing other’s pain compared to healthy controls [88,89]. 

Finally, regarding family functioning, previous studies have evidenced that a parent–child relationship characterized by warmth and emotional support and in which parents set limits and supervise children’s behaviors exerts a protective role on adolescents’ externalizing problems, addictive behaviors, and antisocial behaviors [35,37,67]. The research examining these associations in emerging adulthood is scarce but, in line with our study, has evidenced a protective effect on the emerging adult’s antisocial personality problems [14,19]. Indeed, although social influences from peer and romantic relationships exert increasing weight during this developmental stage, parents continue to assume, for better or worse, a significant role in children’s lives, remaining a key source of social and material support [18,30]. Moreover, even when the emerging adult is no longer living in the family and under direct parental behavioral control, the messages of parental control experienced during adolescence may, in turn, be internalized, still exerting effects on young adults and preventing antisocial personality problems [15].

The protective role of parental behavioral control on emerging adults’ antisocial personality problems was also confirmed by our parallel mediation model. Indeed, both direct and total effects were significant. Moreover, as expected, parental behavioral control predicted low levels of motor impulsivity (that in turn predicted high antisocial personality problems) and high levels of empathic concern (that in turn predicted low antisocial personality problems). The relationships between parental behavioral control and antisocial personality problems via motor impulsivity and empathic concern were also significant. These findings have supported the recent evidence on the complex relationship between emerging adults’ perception of their family functioning, impulsivity problems, empathy functioning, and psychological difficulties [39,68], including personality disorder symptoms [66]. Recently [44], the same findings were also shown in relation to adolescent’s antisocial behaviors. However, to our best knowledge, this is the first study that has explored the possible role played by these associations on emerging adults’ ASPD symptoms. In line with our results, previous studies have shown that the lack of parental behavioral control is prospectively associated with children’s antisocial personality problems [14,15,16,17], impulsivity [90,91], and empathetic impairment [35,67], which international literature has widely shown to be risk factors for antisocial personality problems [48,49,50,51,52,53,54,55,56]. Beyond the direct effect of parental behavioral control on antisocial personality problems, our findings have also supported the recent evidence on indirect effects through its protective influence on impulsivity and empathy [44]. However, the direct effect was greater than indirect effects, suggesting that emerging adults’ motor impulsivity and empathic concern only partially mediate the impact of parental behavior on antisocial personality problems.

### Limitation, Strength, and Implications

There are some limitations to the current study. We conducted an online convenience sampling to collect the data, which does not produce representative results generalizable to the population as a whole. Further studies should use probability sampling techniques. In addition, we evaluated emerging adults’ antisocial personality problems, family functioning, impulsivity, and empathy through self-report instruments. Although these tools are widely validated and extensively used in the field of developmental psychopathology research, further studies should assess these variables using more robust and objective measures (e.g., clinical interviews). Moreover, the instrument we used for the assessment of emerging adults’ family functioning allows obtaining a measure of perceived family functioning. Consequently, information provided may be influenced by emerging adult’s perception biases and should be interpreted with caution. Furthermore, this was a cross-sectional study, which implies considering the causal nature of the relationships that emerged with caution. Subsequent longitudinal studies are needed to confirm our findings. Moreover, although many relevant variables have been included, we have not considered the possible role played by other individual and relational variables that studies have shown to be associated with antisocial personality problems in emerging adulthood (e.g., genetic vulnerabilities, alexithymic characteristics, childhood traumatic experiences, peer influence) [54,92,93]. Notwithstanding the above limitations, the present study has several strengths. This study was the first study to explore the complex relationship between emerging adults’ antisocial personality problems, family functioning, impulsivity, and empathy. Moreover, our sample included both male and female emerging adults, evidencing no significant sex differences in antisocial personality problems. Conversely, historically, the majority of studies have focused almost exclusively on male samples, given that a higher rate of ASPD is generally reported among males than females. However, recent evidence has increasingly shown an increment in antisocial personality problems also among females, suggesting the importance of focusing on both sexes for a better understanding of the phenomenon. 

## 5. Conclusions

Overall, this study has supported the importance of considering the complex relationship between the quality of family functioning, empathy, and impulsivity traits in studying antisocial personality problems in emerging adulthood. Specifically, our results suggested a key role played by parental behavioral control that exerted its protective influence on antisocial personality problems both directly and via motor impulsivity and empathic concern. Although further longitudinal studies using probability sampling techniques are needed to provide higher statistical power and support our preliminary results, our findings have various clinical and public health implications. Indeed, our findings could be informative for the planning of more targeted and effective intervention treatments, supporting the recent evidence that parental involvement in prevention and treatment programs is a critical factor in ensuring success in people of this stage [14]. Moreover, this study has also suggested that the planning of interventions focused on the improvement of self-control and affective empathy should be promoted [94,95].

## Figures and Tables

**Table 1:** Sample demographic characteristics.

Age in years, M (SD)		22.16 (2.18)
Gender, n (%)		
	Male	166 (47.4)
	Female	184 (52.6)
Romantic relationship status, n (%)		
	Single	133 (38)
	Partnered	137 (39.1)
	Cohabitant	62 (17.7)
	Married	18 (5.1)
Living status, n (%)		
	Living with family members	245 (60)
	Not living with family members	105 (30)
Level of education, n (%)		
	Less than high school	30 (8.6)
	High school	155 (44.3)
	More than high school	165 (47.1)
Employment status, n (%)		
	Unemployed	57 (16.3)
	Unemployed student	107 (30.6)
	Employed student	30 (8.6)
	Part-time employment	87 (24.9)
	Full-time employment	69 (19.7)

**Table 2:** Descriptive statistics and Pearson correlation coefficients between the starting theoretical model variables.

	1	2	3	4	5	6	7	8	9	10	11	12	13	14
1.ASR_Antis	1													
2. FAD_PS	−0.05	1												
3.FAD_Com	−0.05	0.46 **	1											
4.FAD_Rol	−0.34 **	0.30 **	−0.09	1										
5.FAD_AI	−0.49 **	0.31 **	−0.20 **	0.53 **	1									
6.FAD_AR	−0.36 **	0.08	−0.08	0.38 *	0.42 **	1								
7.FAD_ BC	−0.63 **	0.06	0.12 *	0.34 *	0.53 **	0.41 **	1							
8.IRI_PT	−0.49 **	−0.04	0.04	0.13 *	0.27 **	0.14 **	0.37 **	1						
9.IRI_FS	−0.23 **	0.04	0.11 *	0.02	0.10 *	0.08	0.26 **	0.36 **	1					
10.IRI_PD	0.06	0.15 **	0.04	−0.16 **	−0.15 **	−0.15 **	−0.10	0.01	0.31 **	1				
11.IRI_EC	−0.53 **	−0.04	−0.02	0.15 **	0.28 **	0.16 **	0.34 **	0.59 **	0.44 **	0.10	1			
12.BIS_Att	0.43 **	0.01	−0.01	−0.19 **	−0.23 **	−0.20 **	−0.26 **	−0.28 **	−0.04	0.10	−0.28 **	1		
13.BIS_Mot	0.59 **	−0.01	0.01	−0.23 **	−0.39 **	−0.26 **	−0.43 **	−0.41 **	−0.12 *	0.01	−0.40 **	0.56 **	1	
14.BIS_NP	0.44 **	0.04	−0.02	−0.17 **	−0.24 **	−0.15 **	−0.31 **	−0.43 **	−0.24 **	0.03	−0.36 **	0.43 **	0.56 **	1
M	6.63	14.28	19.22	24.94	19.32	15.56	24.38	18.82	16.42	12.24	19.36	16.26	20.14	24.26
DS	6.62	3.01	2.79	3.39	3.52	2.70	3.36	4.46	4.56	4.42	4.29	3.33	4.51	4.29

*Note*. ASR = Adult Self Report; FAD = Family Assessment Device; IRI = Interpersonal Reactivity Index; BIS-11 = Barratt Impulsiveness Scale; Antisoc: Antisocial Personality Problem; PS = Problem Solving, Com = Communication, Rol = Roles, AI = Affective Involvement; AR = Affective Responsiveness, PT = Perspective Taking, FS = Fantasy, EC = Empathic Concern, PD = Personal Distress, BC = Behavioral Control, Att = Attentional Impulsivity, Mot = Motor Impulsivity, NP = Non-planning Impulsivity. * *p* < 0.05, ** *p* < 0.01.

**Table 3:** Results of hierarchical regression analyses predicting emerging adults’ antisocial personality problems.

			Adjusted Coefficients		
			*B (SE)*	*t*	*p*
**Covariates**					
	Gender ^a^		0.05 (0.45)	0.18	0.90
	Age		0.12 (0.10)	1.19	0.23
	Relationship status ^b^				
		Partnered	−1.36 (0.52)	−2.61	0.009 **
		Cohabit	−1.29 (1.00)	−1.28	0.20
		Married	−0.75 (1.30)	−0.57	0.56
	Living setup ^c^		−0.28 (0.96)	−0.29	0.71
	Level of education ^d^				
		High school	−0.29 (0.81)	−0.36	0.71
		More than high school	−0.89 (0.86)	−1.04	0.29
	Occupation ^e^				
		Unemployed student	−1.34 (0.69)	−1.94	0.06
		Employed student	−1.15 (0.96)	−1.19	0.23
		Employed part time	−1.83 (0.83)	−2.20	0.03 *
		Employed full time	−2.74 (0.90)	−3.02	0.003 **
**Predictors**					
	FAD	Roles	−0.07 (0.07)	−1.03	0.30
		Affective Involvement	−0.11 (0.08)	−1.34	0.18
		Affective Responsiveness	−0.14 (0.09)	−1.51	0.13
		Behavioral Control	−0.54 (0.08)	−6.27	0.000 ***
	IRI	Perspective Taking	−0.11 (0.06)	−1.77	0.08
		Fantasy	0.06 (0.05)	1.27	0.20
		Empathic Concern	−0.34 (0.06)	−5.07	0.000 ***
	BIS-11	Attentional Impulsivity	0.14 (0.08)	1.79	0.07
		Motor Impulsivity	0.26 (0.07)	3.73	0.000 ***
		Non-planning Impulsivity	0.06 (0.06)	1.02	0.30
Adjusted R^2^					0.64

Note. ^a^ Female is the reference group, ^b^ single is the reference group, ^c^ living with family members is the reference group, ^d^ less than high school is the reference group, ^e^ unemployed is the reference group; SE = Standard error. * *p* < 0.05, ** *p* < 0.01, *** *p* < 0.001.

**Table 4:** Indirect effects of behavioral control on antisocial personality problems through motor impulsivity and empathic concern.

Indirect Effect	Effect (BootSE)	LLCI	ULCI
Total	−0.37 (0.05)	−0.49	−0.27
Behavioral Control→Empathic Concern→Antisocial Personality Problems	−0.15 (0.03)	−0.23	−0.09
Behavioral Control→Motor Impulsivity→Antisocial Personality Problems	−0.22 (0.04)	−0.31	−0.14

## Data Availability

Data available on request to the authors.
